# Isotope analyses to explore diet and mobility in a medieval Muslim population at Tauste (NE Spain)

**DOI:** 10.1371/journal.pone.0176572

**Published:** 2017-05-04

**Authors:** Iranzu Guede, Luis Angel Ortega, Maria Cruz Zuluaga, Ainhoa Alonso-Olazabal, Xabier Murelaga, Miriam Pina, Francisco Javier Gutierrez, Paola Iacumin

**Affiliations:** 1 Department of Mineralogy and Petrology, Faculty of Science and Technology, University of the Basque Country-UPV/EHU, Vizcaya, Spain; 2 Department of Stratigraphy and Palaeontology, Faculty of Science and Technology, University of the Basque Country-UPV/EHU, Vizcaya, Spain; 3 “El Patiaz” Cultural Association, Cuesta de la Cámara 12, Tauste, Zaragoza, Spain; 4 Department of Physics and Earth Sciences, University of Parma, Parma, Italy; University of Otago, NEW ZEALAND

## Abstract

The Islamic necropolis discovered in Tauste (Zaragoza, Spain) is the only evidence that a large Muslim community lived in the area between the 8th and 10th centuries. A multi-isotope approach has been used to investigate the mobility and diet of this medieval Muslim population living in a shifting frontier region. Thirty-one individuals were analyzed to determine δ^15^N, δ^13^C, δ^18^O and ^87^Sr/^86^Sr composition. A combination of strontium and oxygen isotope analysis indicated that most individuals were of local origin although three females and two males were non-local. The non-local males would be from a warmer zone whereas two of the females would be from a more mountainous geographical region and the third from a geologically-different area. The extremely high δ^15^N baseline at Tauste was due to bedrock composition (gypsum and salt). High individual δ^15^N values were related to the manuring effect and consumption of fish. Adult males were the most privileged members of society in the medieval Muslim world and, as isotope data reflected, consumed more animal proteins than females and young males.

## Introduction

Muslims invaded most of the Iberian Peninsula in the Early Middle Ages (AD 711) and remained for the next seven centuries, until 1492 when the Christian Kingdoms totally reconquered the peninsula. The northern frontier of the country captured by the Muslims, known as al-Andalus, extended eastward on the southern slopes of the Cantabrian range from the present Galicia to Catalonia. Following the Muslim conquest, al-Andalus was at first (711–750) a province of the Umayyad Caliphate centered on Damascus. From 740 a series of civil wars between various Muslim groups resulted in the breakdown of the Arab empire and the Emirate of Cordova (c. 750–929) emerged. In 929 the emir of Cordova proclaimed himself Caliph and the period of the Caliphate of Cordova was established (929–1031). The Cordova Caliphate collapsed during a civil war and Al-Andalus broke up into a number of mostly independent states called taifa kingdoms. The independent taifas were too weakened to defend themselves against the Christian Kingdoms in the north and west, allowing the Reconquest. The Christian reconquest of Iberia ended with the final assault on the Emirate of Granada in 1492. From 711 to 1492, as political dominions changed, the boundaries between the Christian north and the Islamic south shifted constantly.

In the Ebro Valley, the first Muslims arrived in the early 8th century conquering the main towns without any relevant or attested resistance and the Upper March (or northern frontier) was established along the Ebro basin. Thus began the Muslim period in the Ebro Valley that, for four centuries, was centered on the metropolis of Saragossa. Shortly after the Muslim conquest, the nobleman Count Cassius converted to Islam to preserve his lands and political power and founded the Banu Qasi dynasty. In the 9th century the Upper March was under the dominion of the Banu Qasi dynasty as a semi-autonomous territory within the Cordova caliphate [1,2]. During the 9th century, the Banu Qasi lineage was successively loyal and rebellious toward the Cordova emir. In the second half of the century, the Banu Qasi domains increased considerably, extending north to the Pyrenees and east nearly to the Mediterranean coastline. However, in the later 9th century the Cordova emir recovered most of the Upper March territories and in the early 10th century, harassed by its Christian neighbors and without the support of Cordova, the Banu Qasi dynasty lost all its territories [3].

The society of Al-Andalus was made up of three main religious groups: Christians, Muslims and Jews, who inhabited distinct neighborhoods in the cities. Islamic society stratification was mainly by ethnic division. The kinship system ascribed importance only to relationships through males and endogamous marriages were viewed as the ideal system [4]. The more powerful a tribal group was, the more women it would attract from outside and the fewer it would lose, and the more endogamous it would become. Under Islamic law, the most privileged members of society were devout Muslim men, and women were treated as second-class citizens [4]. In particular, women’s rights were contingent on their place within society on several levels, including their religious, economic and marital status. Under Islamic law, other groups in society such as Jews and Christians had fewer rights and privileges, to varying degrees.

Within this framework, Tauste was placed midway between the two most significant cities: Saragossa, metropolis of the Upper March, and Tudela, the center of the Banu Qasi territory.

The Muslim occupation of Tauste (Zaragoza, Spain) has been considered incidental and even non-existent, according to traditional and written sources. However, recent excavations suggest a large stable Muslim population lived in the town from the early Islamic period in the Iberian Peninsula. In 2010, a cemetery with several human skeletons aligned perpendicular to Mecca was discovered. The bodies were placed on their right side, facing towards Mecca, as is characteristic of a Muslim cemetery [5]. In contrast, no remains of the Muslim village associated with this necropolis have yet been found. Multi-isotopic studies, including radiogenic strontium, stable oxygen, carbon, and nitrogen, have been used to reconstruct the geographic origin, mobility and dietary practices of the Tauste individuals during the Islamic period in the Iberian Peninsula. Stable isotope composition of bone collagen reveals information about nutrition, life history, and mobility in past populations [6–10].

### Isotope analyses in bioarchaeology

The analysis of carbon and nitrogen isotope composition in bone collagen constitutes an approach to palaeodietary reconstruction. It can provide information about the protein portion of the diet averaged over roughly the last 10 years prior to death and also about different protein sources [11,12].

Carbon isotope analysis provides information about the ecosystem that foodstuffs come from, distinguishing between terrestrial and marine ecosystems. In the case of a terrestrial diet, it informs about the plants that were consumed. Two classes of plants are distinguished according to their photosynthetic pathways: C_3_ plants and C_4_ plants. C_3_ plants are most vegetables, wheat (*Tritium*) and barley (*Hordeum vulgare*), while C_4_ plants include millet (*Pennisetum*), maize (*Zea mays*) and sugar cane (*Saccharum officinarum*). C_4_ plants exhibit more enriched carbon values than C_3_ plants, so that the mean δ^13^C values are -13‰ and -27‰ respectively [13,14]. Marine plants are all C_3_ plants and their average values are about 7.5‰ higher than terrestrial C_3_ plants. Carbon isotope composition can be used to distinguish marine protein consumption in terrestrial C_3_-based diets, but when C_4_ plants are involved marine and terrestrial values can overlap [15,16]. Carbon fractionates in δ^13^C by only about 1‰ throughout the food chain [6–8,17]. In freshwater ecosystems the δ^13^C composition of plants is variable and consequently freshwater fish exhibit a broad range of δ^13^C values that are largely depleted [18,19]. Therefore, δ^13^C ratios more negative than -22‰, the value corresponding to the low-end of a diet based only on C_3_ terrestrial plants, suggest freshwater fish consumption.

Nitrogen isotope values reflect the intake of animal proteins and inform about the trophic level of an individual [10,20,21]. Thus, nitrogen isotopes in terrestrial ecosystems are enriched in δ^15^N by 2–5‰ (on average, 3‰) from food to body tissue as trophic levels increase [8,22,23] Terrestrial protein sources have δ^15^N values ranging from 5‰ to 12‰, while aquatic food sources range from about 12‰ to 22‰ for marine fish and 7.2‰ to 16.7‰ for freshwater fish [24–28]. When C_3_ plants are consumed, nitrogen isotope analysis is combined with carbon isotope analysis to distinguish between proteins derived from terrestrial, freshwater and marine resources. Other reasons for variability in δ^15^N ratios of plants and animals include natural environmental conditions such as salinity and aridity or anthropogenic factors like manuring [29,30]. In general, human diet corresponds to a mixture of food with different isotope signatures. Plots of collagen δ^13^C vs δ^15^N values can be interpreted as mixtures of multiple components [31,32] that do not yield unique solutions, but may outline the dominant components in the diet of the studied individuals. Besides, stable nitrogen isotope analysis can also be used to investigate breastfeeding and weaning practices. In fact, during breastfeeding, children exhibit δ^15^N values enriched about 2–3‰ over that of their mothers [33]. Strontium and oxygen isotopes are two independent isotopic systems in which strontium reflects local geology and oxygen reflects geography and can be used to reconstruct movements of past populations. The combination of these two isotopic systems is able to constrain possible areas and provide information about an individual’s area of origin and thus determine mobility patterns [34–36].

Oxygen and strontium are fixed in phosphate in teeth and bones through ingested food and water. Strontium isotopes appear by substituting calcium in biogenic phosphate [37–39]. After formation during infancy, tooth enamel does not incorporate other elements and thus will reflect the geological composition of the place of residence during childhood, assuming that childhood residence and food production area coincide, at least for the majority of the food intake [37,40]. However, these patterns are not perfectly predictable at any level, because of vagaries in available food over time, and because the strontium ratio reflects an average value that synthesizes the geological composition of the different food provenances ingested during childhood. The average expected patterns are used to predict the most likely geographic links between tissue and location. In contrast, bones are continuously remodelled throughout an individual’s lifetime.

The radiogenic strontium isotopes are related to geology and vary according to the composition and age of bedrock. The strontium concentration in organisms varies according to the trophic level but the ^87^Sr/^86^Sr isotope signature of humans and fauna has negligible metabolic fractionation and will reflect the isotope signature of the underlying bedrock [39,41–48]. ^87^Sr/^86^Sr ratios in bedrock, soils, water and plants will be reflected in humans and animals that consume food and water from those sources [21,38,49]. Since the ^87^Sr/^86^Sr isotope ratio is inherited from the local environment, it is necessary to define the local bioavailable strontium isotope signature to evaluate residential mobility of individuals. There are several methods to establish the local baseline of the isotope signature by analyzing environmental samples including freshwater, soil leachates, ancient fauna and present-day small wild animals [38,50–52]. However anthropogenic activities such as the use of fertilizers could modify the strontium isotope ratios of modern ecosystems [53–56].

In contrast, the oxygen isotope reflects the isotopic composition of ingested water that is derived from meteoric water. The δ^18^O in precipitation varies regionally according to temperature and other climatic parameters, such as distance from the coastline, altitude and latitude [57–60]. Oxygen isotopes in the body are subject to several steps of metabolic fractionation. The fractionation mechanisms are relatively well known, allowing the calculation of approximate drinking water (δ^18^O_w_) values from the δ^18^O_p_ of biogenic phosphate by means of conversion equations [57,60–66]. Despite complexities in the calculation of meteoric water isotope composition in the past, the oxygen isotope composition of human remains allows the identification of palaeomobility patterns.

The aim of this study was to reconstruct palaeomobility and palaeodiet patterns in the medieval Muslim population at Tauste. Tauste Muslim necropolis constitutes a suitable site to examine human mobility since it was located on the northern frontier during a very convulsive period of time. In addition, the palaoedietary pattern can illustrate the basic dynamics of medieval Muslim social life. For these purposes, stable isotopes (δ^13^C, δ^15^N, δ^18^O) and radiogenic strontium (^87^Sr/^86^Sr) were investigated to obtain information about nutrition and social stratification.

## Archaeological setting

**Fig 1:**
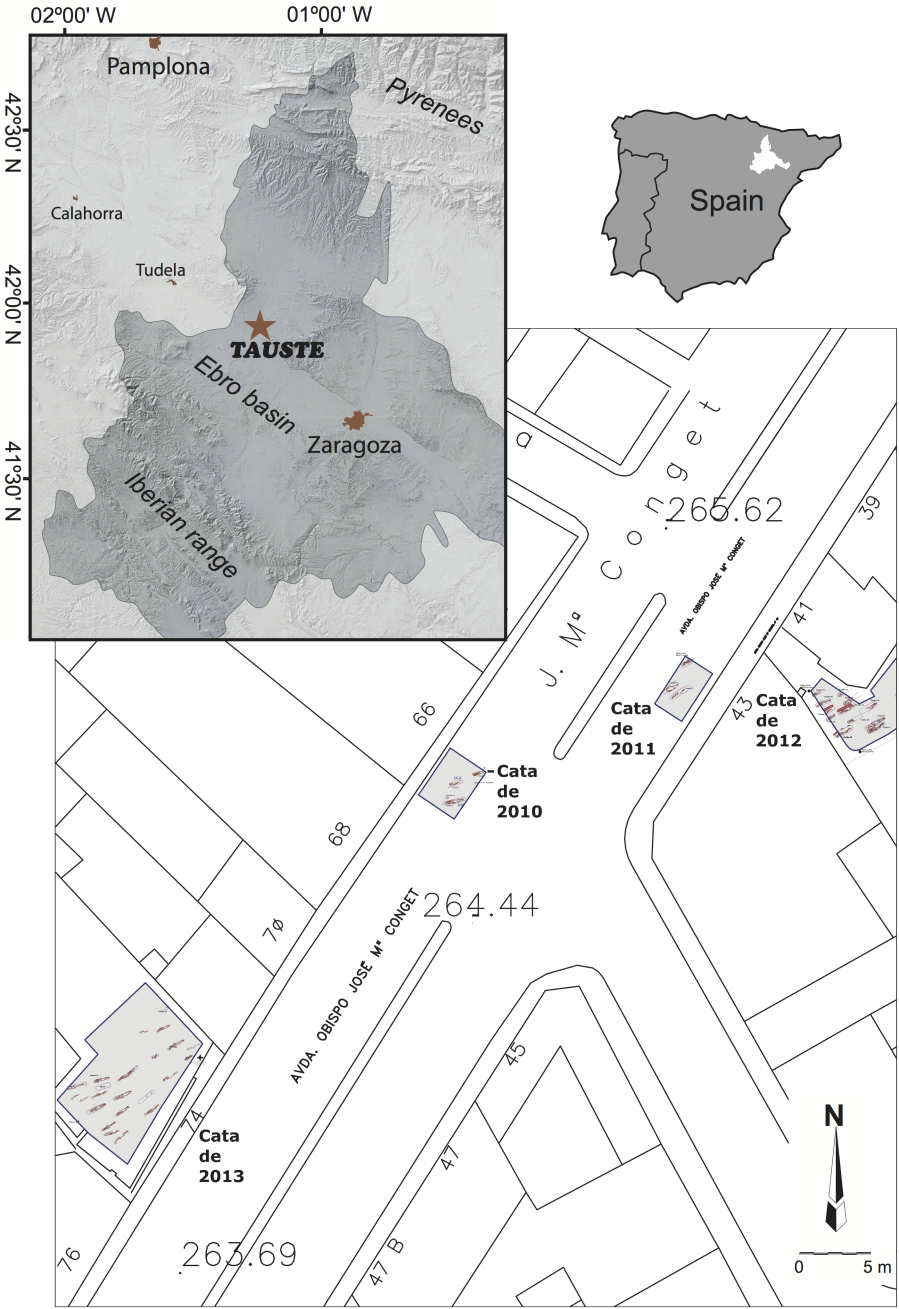
Location of Tauste archaeological site and the excavated areas. Reprinted from under CC by license, with permission from [Instituto Geográfico Nacional (IGN)], original copyright [2015].

**Fig 2:**
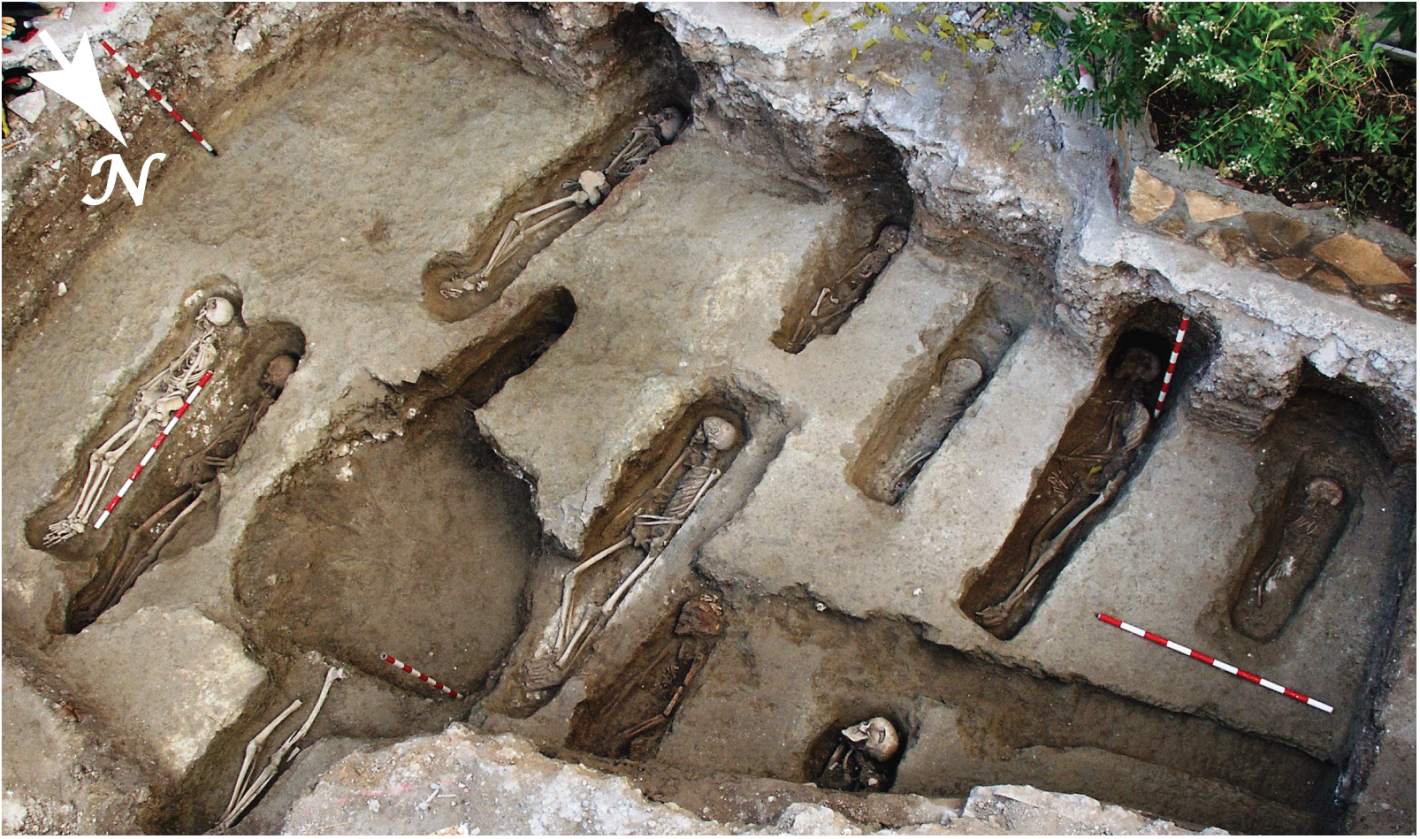
Aerial view of some burials showing individuals placed in the graves following the Muslim burial rituals (facing east).

Tauste archaeological site is located in the town with the same name in the province of Zaragoza (northern Spain) (Fig 1). Tauste is in the Ebro basin, on the River Arba, a tributary of the River Ebro. The Muslim archaeological site of Tauste is formed only by the cemetery, with a total absence of other vestiges of Islamic population. All the graves were aligned SW-NE and the human bodies were carefully placed on their right side, facing Mecca, indicative of a Muslim necropolis [67] (Fig 2). All individuals were found in anatomical connection. Graves were dug in clay soil without any structure, or only a minimum structure formed by rammed earth on the sides according to Muslim burial rituals. More complex tombs corresponding to double grave burials (*shaq* or *ladj*) were found. A similar burial system has been documented in other Muslim necropolises in the Iberian Peninsula, such as Marroquíes Bajos (Jaen) [68], Tossal de Manises (Alicante) [69] or the recent find at Valdeherrera (Calatayud). The human remains extended over an area of two hectares and the density of graves (0.25–0.30 individuals/m^2^) indicates a minimum of 4.500 burials, excluding children [70,71]. Only a simple bronze hoop earring was found in a female’s grave, and the lack of grave goods is also indicative of Islamic funeral rituals. The excavations have found at least two levels of burials, indicating this cemetery was in use during an extended period of time.

**Fig 3:**
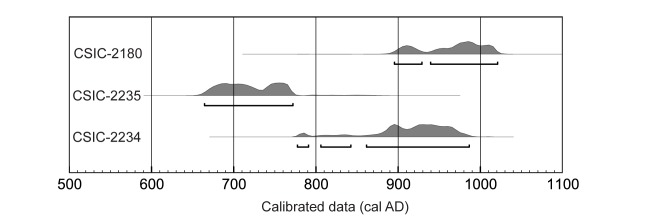
Radiocarbon dating of human bone samples from Tauste calibrated with OxCal v4.2.4 [72] and IntCal13 atmospheric data [73].

Radiocarbon dating of human bones dates the graveyard in the 8th to 10th centuries and it could be one of the oldest Muslim necropolises in the Iberian Peninsula (Table 1, Fig 3). Calendar ages were determined using the Oxcal v 4.2.4 program [72] with the latest IntCal13 calibration curve for atmospheric data [73]. Calibrated age ranges correspond to 95.4% probability (2σ) and are expressed in years cal AD. The age and extent of the necropolis suggest Tauste was a thriving village in the times of the Banu Qasi dynasty, when the northernmost limit of Al-Andalus was established [74,75].

**Table 1:** Calibrated radiocarbon dating of the Tauste site.

Sample	Lab. Code	Age BP	Age cal AD		
			from	to	%
Tomb 1	CSIC-2180	1072 ±32	895	929	22.7
			939	1021	72.7
Tomb 2	CSIC-2235	1286±31	664	772	95.4
			777	791	3.3
Tomb 3	CSIC-2234	1133±28	806	842	5.7
			861	986	86.4

**Fig 4:**
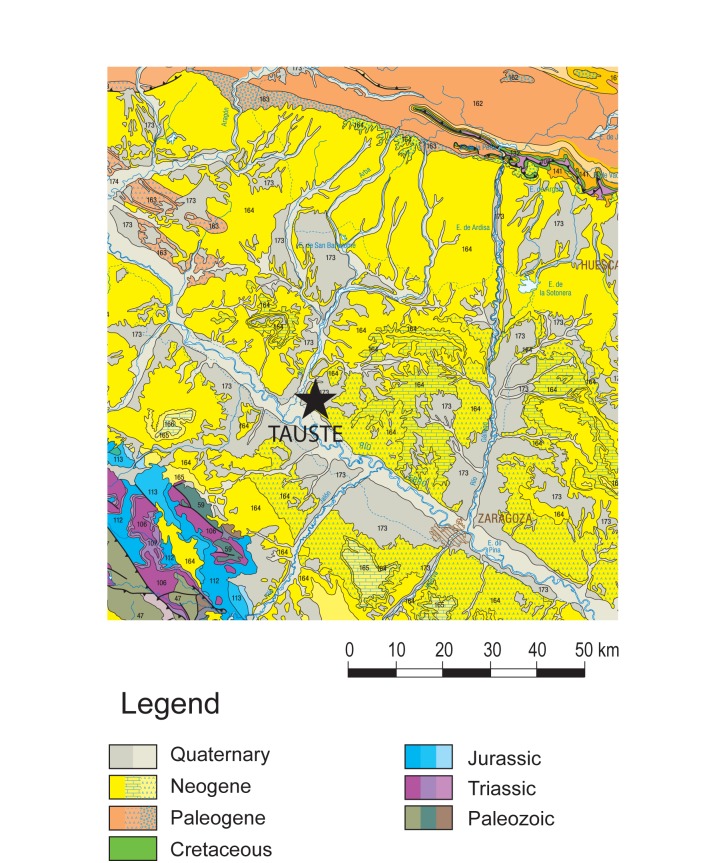
Geological map of the Tauste region showing evaporitic nature of the bedrock. Reprinted from [76] under CC by license, with permission from [Instituto Geológico y Minero de España (IGME)], original copyright [2015].

Geologically, the Muslim necropolis of Tauste is located in the Ebro Basin, composed by Tertiary (Miocene) and Quaternary sedimentary rocks of continental origin (Fig 4) [76]. Miocene materials around Tauste are composed by claystones with interbedded gypsum layers. These materials correspond to evaporite lacustrine facies, i.e. sediments deposited in the centre of a continental sedimentary basin. Miocene deposits are overlaid by Quaternary materials consisting mainly of river terrace deposits and fluvial sediments. The evaporitic nature of the bedrock causes a large increment in salt contents in the environment. In fact, high levels of sodium chloride and sulphate ions have been found in the freshwater River Arba [77,78].

## Materials and methods

This study deals with archaeological skeletal material and all necessary permits were obtained for the described study, which complied with all relevant regulations. The excavation licenses were issued by the General Director of Cultural Heritage of the *Gobierno de Aragon* (Spain) and are stored in its archives. Following excavation campaigns 2010/2013, the bones and teeth samples were transferred to the Heritage and Cultural Landscape Research Group (GIPyPAC) at the University of Basque Country-UPV/EHU, Spain for investigation. At present all archaeological remains, including the human bones, recovered at the site of Tauste are stored in the Museo de Zaragoza.

**Fig 5:**
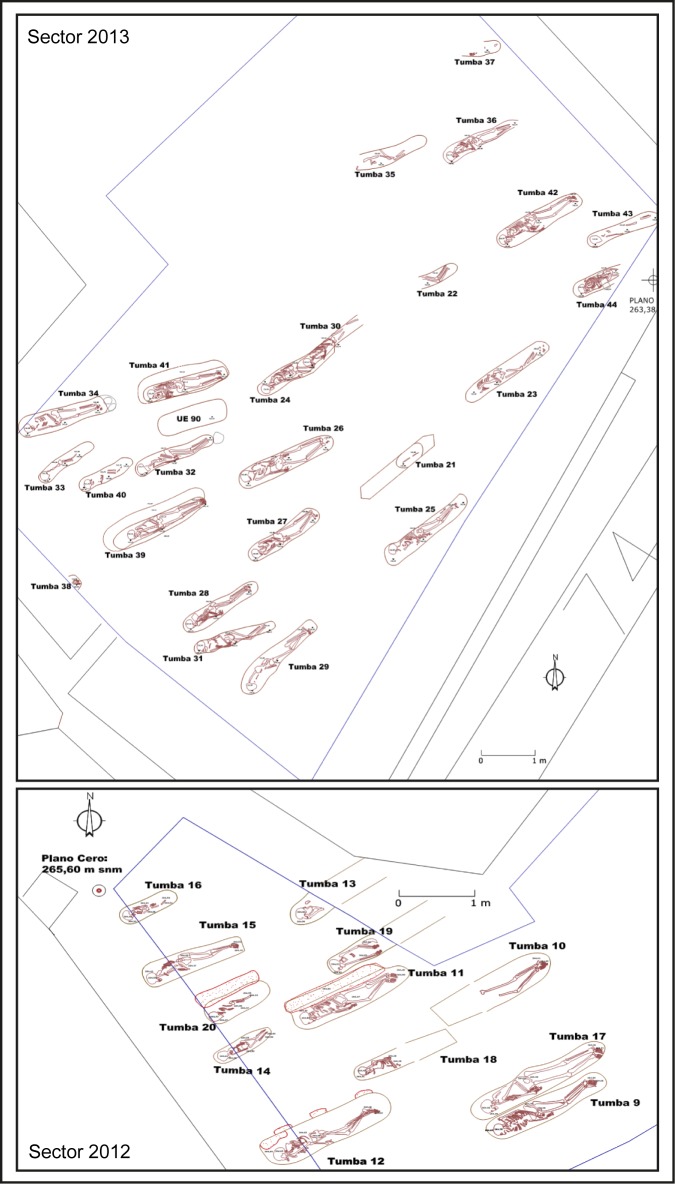
Detailed map of two excavated areas (sectors 2012 and 2013) showing studied individuals.

Carbon and nitrogen isotope measurements have been performed in bone collagen extracted from 31 human individuals corresponding to the sectors excavated in 2012 and 2013 (Fig 5) and nine faunal bone samples. Additionally, 23 teeth and 8 bone samples were analyzed for strontium and oxygen isotope studies. In order to define the strontium isotope baseline, four soil samples and one freshwater sample were analyzed. The soil samples were collected in different parts of the cemetery, while the freshwater sample was collected from the River Arba near Tauste. Surface waters were collected from the banks of the river. Before analysis, water samples were filtered to remove suspended particles.

Water aliquot was filtered with disposable syringe filters (0.45 um) and 10 mL aliquot was transferred to a 15 mL Teflon (Savillex™) vial, evaporated down on a hot plate at 80°C overnight and then dissolved in 1.5 mL of 2N HNO_3_.

The measured individuals were corresponded to 12 males, 10 females and 9 of indeterminate sex [71]. The individuals were categorized by age into infants (0–3 years), children (6–12 years), juveniles (12–17 years), young adults (18–34 years), middle-aged adults (35–50 years), and older adults (older than 50 years). According to these categories, 2 individuals are older adults, 11 are middle-aged adults, 6 are juveniles, 7 are young adults, 4 are infants and 1 is of indeterminate age. Sex determination was carried out according to the classical patterns of dimorphism and age was defined by the most reliable markers: changes in auricular surface and pubic symphysis, epiphyseal closure, cranial sutures and dental eruption [79]. The faunal samples corresponded to three wood mice (*Apodemus sylvaticus*), three shrews (*Crocidura russula*) and two common barbels (*Barbus barbus*) and a madrilla (*Parachondrostoma miegii*).

For carbon and nitrogen isotope analyses, bone collagen was extracted following the procedure in Bocherens et al. [80]. 300 mg of bone sample powder were demineralised in 1M HCl for 20 min at room temperature until the sample dissolved. To remove humic acid the samples were rinsed with distilled water and treated with 0.125 M NaOH. The resulting insoluble fraction after being rinsed again with distilled water was gelatinized in HCl solution at pH3 for 17 h at 90°C. Then, samples were filtered with disposable syringe filters (5 um), freeze-dried and finally lyophilized. Lyophilized collagens (2.5–3.5 mg) were enclosed in tin capsules for isotopic analysis. Carbon and nitrogen isotope analyses were performed using an elemental analyzer on line with a continuous-flow isotope ratio mass spectrometer (EA-IRMS) at Iso-Analytical (Cheshire, UK). Replicate measurements of the liver standard NBS-1577B and ammonium sulphate IA-R045 working standard were run to confirm instrument accuracy. Replicate analysis of the NBS-1577B δ^13^C standard during runs gave a ^13^C/^12^C of −21.62 ± 0.02 (1σ, n = 7) and ^15^N/^14^N of 7.62 ± 0.13 (1σ, n = 7), and the IA-R045 working standard during runs gave a ^15^N/^14^N of −4.56 ± 0.17 (1σ, n = 4) and ^13^C/^12^C of −26.1 ± 0.03 (1σ, n = 4). Isotopic values are reported as δ values in per thousand (‰) relative to international defined standards for carbon (VPDB: Vienna Pee Dee Belemnite) and nitrogen (AIR: Ambient Inhalable Reservoir). The instrumental precision for δ^13^C was ± 0.06‰ or better and for δ^15^N was between ± 0.06‰ and ± 0.08‰, determined by replicated analyses of internal standards.

Tooth enamel and bones were used to determine strontium and oxygen isotope composition. The samples were washed in an ultrasonic bath to remove impurities and further cleaned by mechanical abrasion to remove the outer surface and avoid potential contamination.

For strontium isotope analysis a fraction of dental enamel was collected mechanically with a diamond-coated trepanation drill (MF-perfect, W & H Dentalwork, Bürmoos, Austria). The enamel sample was taken transversally. Enamel and bone samples (~10 mg) were dissolved in 7 mL Savillex® vials (Minnetonka, MN, USA) with 1.5 mL of 2N HNO_3_ (analytical grade purified by subboiling distillation). In order to establish the local isotopic composition, two water samples and four soil samples were also analyzed. 15 mL of freshwater was evaporated to dryness and then dissolved in 2 mL HNO_3_. A 1g aliquot soil sample was leached by adding 2.5 mL 1 M ammonium nitrate (NH_4_NO_3_) and shaking for 8 h to obtained the bioavailable Sr. After samples were centrifuged at 3000 rpm for 15 min, the supernatant was extracted (~1–2 mL) and evaporated to dryness and then redissolved in 2 mL HNO_3_. The solutions were loaded into cation exchange columns filled with Sr.spec® (ElChroM industries, Dariel, IL, USA), a strontium selective resin. The resin was used once to elute the sample and then discarded. Strontium procedural blanks were less than 100 pg and hence provided a negligible contribution.

The radiogenic strontium isotope samples were analyzed on a Neptune multi-collector inductively coupled plasma mass spectrometer (MC-ICP-MS) at the Advanced Research Facilities (SGIker) of the University of the Basque Country (UPV/EHU). ^87^Sr/^86^Sr measurements were corrected for krypton (Kr) and rubidium (Rb) interferences and normalized for instrumental mass bias using ^87^Sr/^86^Sr = 8.375209. Repeated analyses of NIST SRM-987 international standard yielded a value of ^87^Sr/^86^Sr = 0.710262 ± 0.000026 (2σ, n = 3). Long-term ^87^Sr/^86^Sr value, determined over a twenty-two month period, was 0.710266 ± 0.000021 (2σ, n = 47).

Tooth enamel was also prepared for oxygen isotope analysis following the procedure described in Stephan [81]. 60 mg of dental enamel powder was processed. The organic matter was removed with a solution of 2.5% NaOCl for 24 h at room temperature followed by a 48 h treatment in 0.125M NaOH at room temperature. The hydroxyapatite powder free of organic matter was dissolved in 2 mL of HF for 24 h. The phosphate solution and the residue composed of CaF_2_ were separated by centrifugation, pipetted into a 100 mL glass tube and neutralized with 3 mL 2M KOH. Silver phosphate (Ag_3_PO_4_) was precipitated by adding 30 mL of a buffered silver amide solution (0.2 M AgNO_3_; 1.16 M NH_4_NO_3_; 0.75 M NH_4_OH) gradually warmed up to 70°C, holding the temperature for 5–6 h and cooling down slowly. Silver phosphate crystals were filtered on a weighed 0.2μm filter and washed several times with double distilled water, then dried at 50°C for 1–2 h. Subsequently, 0.3 mg of Ag_3_PO_4_ was mixed with 0.5–1 mg of AgCl and 0.3 mg of graphite in silver capsules. The capsules were transferred into the autosampler carousel of the Temperature Conversion Elemental Analyser (TCEA) and degassed for 30 minutes at 80°C in a vacuum. The oxygen isotope analyses were performed on a Thermo Finnigan TCEA coupled to a Delta Plus XP Spectrometer at the University of Parma. Isotopic compositions were given in the conventional δ-notation relative to V-SMOW (Vienna-Standard Mean Ocean Water). Normalization to the V-SMOW scale was based on four replicated international reference materials provided by the International Atomic Energy Agency (IAEA): IAEA-601, IAEA-602, IAEA-CH6, and IAEA-SO-6. The analytical precision of a single determination was better than ±0.4‰.

To identify outliers in δ^18^O_PO4_ and in ^87^Sr/^86^Sr within the Tauste population two statistical techniques were used. Boundaries of intra-sample variation based on two measurements of scales were defined: ± 2 standard deviation (2SD) from the mean and Tuke’s inter-quartile range method (IQR) considering 1.5xIQR and 3xIQR [82]. Parametric statistics were used to describe isotope distribution and compare isotope values between groups. Differences between sample groups were analyzed by applying an unpaired Student’s t-test. Statistical significance was accepted as p < 0.05. Statistical analysis was performed with SPSS v.20 (Statistical package for Social Sciences).

## Results and discussion

### Residential mobility

Strontium and oxygen isotope data for 23 tooth enamel samples, 8 rib bones and local geological materials to establish the strontium baseline signature at Tauste are shown in Table 2.

**Table 2:** Strontium, carbon, nitrogen and oxygen isotope results for human bones and teeth, and freshwater and soil samples from Tauste.

Sample	Sex	Material	Tooth Type	Age	% N	δ^15^N	% C	δ^13^C	C/N	δ^18^O	^87^Sr/^86^Sr	2SE
T-9	F	Rib		50–60	15.4	12.7	42.7	-19.3	3.23		0.70868	0.00001
		Enamel	M2							16.35	0.70855	0.00002
T-11	M	Rib		45	14.9	15.4	40.8	-19.5	3.19			
		Enamel	M2							16.72	0.70855	0.00002
T-12	M	Rib		40–45	16.7	15.7	45.8	-19.5	3.20			
		Enamel	I							16.78	0.70858	0.00001
T-13	M	Rib		40–50	15.6	16.3	42.8	-19.0	3.21			
		Enamel	M2							18.00	0.70857	0.00002
T-14	?	Rib		2–4	16.6	16.8	45.6	-18.9	3.21			
T-15	F	Rib		33–45	13.9	15.6	39.2	-18.5	3.29		0.70867	0.00001
		Enamel	M2							14.25	0.70860	0.00001
T-16	?	Rib		2	15.2	16.3	42.3	-17.0	3.25			
		Enamel	M2							17.03	0.70863	0.00001
T-17	M	Rib		33–45	15.5	15.3	42.5	-19.3	3.21		0.70868	0.00001
		Enamel	M2							16.65	0.70861	0.00002
T-18	M	Rib		25–35	15.1	13.7	42.1	-18.8	3.24		0.70869	0.00001
		Enamel	M2							17.17	0.70862	0.00001
T-19	F	Rib		33–45	14.5	14.1	40.2	-19.0	3.24			
		Enamel	M2							17.14	0.70850	0.00002
T-21	?	Rib		Indet	15.0	9.6	41.7	-19.3	3.25		0.70869	0.00001
T-22	?	Rib		>17	15.0	15.9	41.3	-19.1	3.22			
T-23	F	Rib		30–35	15.8	14.3	43.2	-19.1	3.20			
T-24	F	Rib		25–35	13.9	16.3	38.5	-19.3	3.23		0.70868	0.00001
		Enamel	M2							17.9	0.70837	0.00001
T-25	F	Rib		>20	15.3	16.0	42.4	-19.0	3.24			
T-26	M	Rib		25–35	15.9	16.0	43.6	-19.4	3.19			
		Enamel	M2							16.64	0.70862	0.00002
T-27	(m)	Rib		15–17	15.1	13.5	41.7	-19.3	3.23			
		Enamel	C							16.92	0.70857	0.00002
T-28	?	Rib		12–15	14.8	15.8	40.9	-19.3	3.22			
		Enamel	M2							17.8	0.70858	0.00002
T-29	?	Rib		>25	13.1	15.5	36.2	-19.1	3.23			
T-30	F	Rib		35–45	15.4	14.3	42.2	-18.4	3.21			
		Enamel	M2							18	0.70864	0.00001
T-31	F	Rib		16–20	15.8	14.2	43.7	-18.8	3.22			
		Enamel	M2							14.48	0.70859	0.00002
T-32	F	Rib		45–55	15.8	10.8	43.6	-19.0	3.23		0.70883	0.00002
		Enamel	M2							17.9	0.70867	0.00002
T-33	?	Rib		4–6	15.7	14.6	43.7	-19.5	3.24			
		Enamel	M2							17.0	0.70864	0.00002
T-34	M	Rib		12–15	15.8	15.5	43.9	-18.7	3.25			
		Enamel	M2							16.9	0.70853	0.00002
T-35	?	Rib		40–50	15.1	16.9	41.9	-19.2	3.25			
T-36	F	Rib		50–65	14.9	13.9	41.4	-19.1	3.24			
T-39	M	Rib		40–50	14.1	16.5	39.2	-19.5	3.23		0.70867	0.00001
		Enamel	M2							17.4	0.70860	0.00002
T-40	?	Rib		3–5	16.1	17.5	44.1	-19.9	3.20			
		Enamel	M2							17.06	0.70866	0.00002
T-41	M	Rib		20–30	16.3	15.0	45.3	-19.5	3.24			
		Enamel	M2							17.0	0.70860	
T-42	M	Rib		35–45	16.7	17.0	46.4	-19.0	3.23			
		Enamel	M2							19.1	0.70851	
T-44	M	Rib		25–35	16.7	14.0	46.2	-18.9	3.23			
		Enamel	M2							19.3	0.70856	
T-39*		Soil									0.70868	0.00002
T-41*		Soil									0.70869	0.00002
T-42*		Soil									0.70867	0.00001
T-44*		Soil									0.70867	0.00001
W-Arba		Freshwater									0.70843	0.00001

m = male; (m) = probably male; M = molar; C = canine; I = incisor;? = undetermined; f = female.

* = Soil samples.

2SE = standard error.

**Fig 6:**
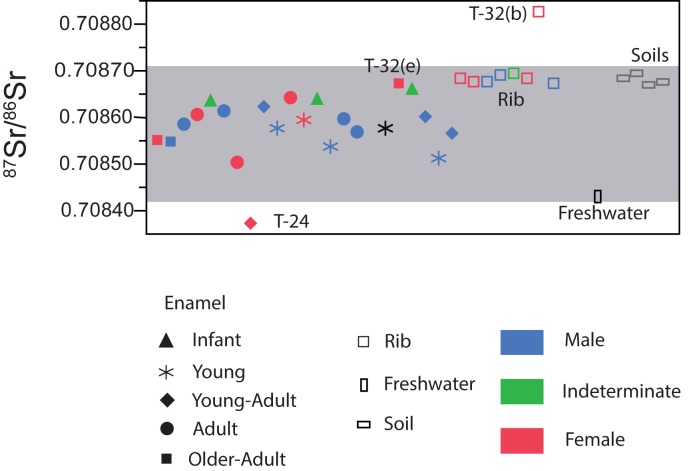
Strontium isotope variation of studied samples. Grey area corresponds to local strontium background defined by local freshwater and soils. Abbreviations (e): enamel sample, (b): bone sample.

To establish local bioavailable strontium, bedrock, fauna, soils and surface water are used but archaeological microfauna or snail shells are considered the most appropriate material [32,43,45,38,83,84]. Since the site is a Muslim necropolis, no fauna remains are associated with the burials. Therefore, to define the Tauste bioavailable strontium isotope baseline, surface water and soils were considered. Local ^87^Sr/^86^Sr isotope composition determined by the local soils varies between 0.70867 and 0.70869, while the freshwater composition is 0.70843. The ^87^Sr/^86^Sr ratios of enamel vary between 0.70837 and 0.70867 and human bone samples range from 0.70867 to 0.70883 (Fig 6).

According to the defined local baseline, most individuals buried in Tauste have a ^87^Sr/^86^Sr signature consistent with local origin. Only two individuals plot outside the estimated local compositional range (1σ) according to outliers identifying by 1.5xIQR and 2SD method. Enamel of young adult female T-24 displays a lower radiogenic strontium isotope value (^87^Sr/^86^Sr = 0.70837) and the rib of adult female T-32(b) presents a higher strontium value (^87^Sr/^86^Sr = 0.70883) (Fig 6). These compositions indicate two different mobility patterns for these females. The female T-24’s strontium value suggests she was born in another place and died in Tauste. Whereas female T-32(b)’s isotope value suggests that she spent her childhood in Tauste, during adulthood moved to another location and came back to Tauste a few years before she died.

**Fig 7:**
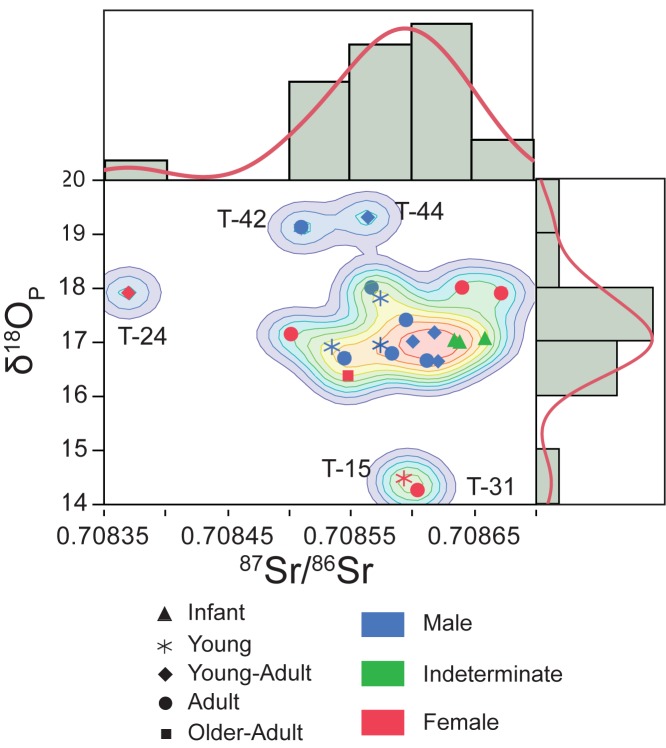
δ^18^O versus ^87^Sr/^86^Sr in the tooth enamel of Tauste individuals. Kernel density contour lines represent 10%.

**Fig 8:**
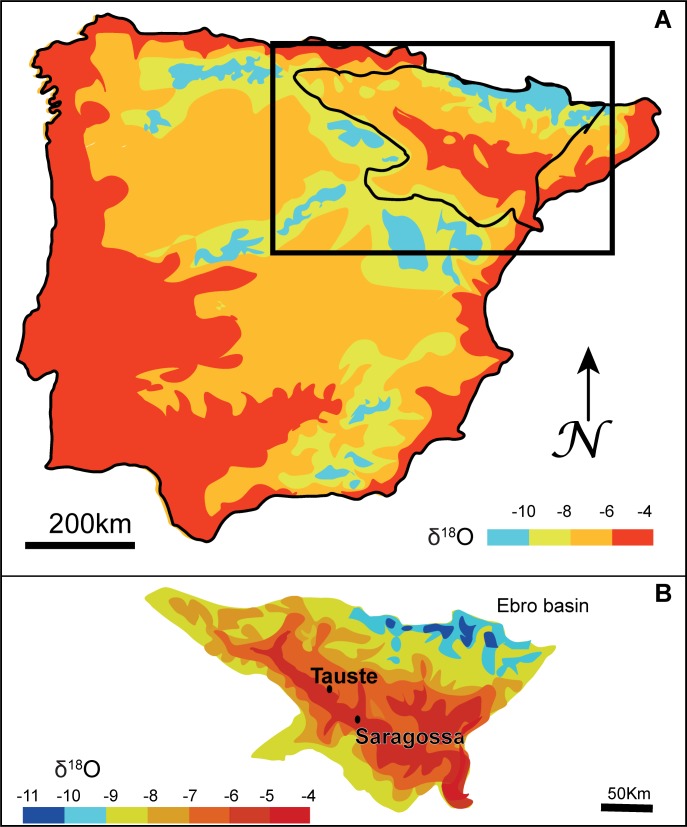
A. Large-scale map of oxygen isotope signatures in the Iberian Peninsula [86,87]. B. Detailed map of oxygen isotope signature of the Ebro Valley [86,87].

The enamel phosphate oxygen ratios (δ^18^O_p_) cover a broad range of values from 14.25‰ to 19.30‰. Based on kernel density estimations (Fig 7), data can be split into three groups: a larger group (n = 18) with δ^18^O_p_ ratios between 16.4‰ and 18‰ and two smaller groups, one formed by two males with δ^18^O_p_ signature higher than 19.2‰ and the other formed by two females with δ^18^O_p_ signature lower than 14.4‰. Local meteoric water δ^18^O_dw_ is -5.6‰ (average values from 2000 to 2006) considering data from Zaragoza airport station [85]. δ^18^O_dw_ values for humans from Tauste were calculated using the available phosphate/drinking water equations [58,60,61,63,66], and comparing them with expected local water values derived from IAEA/WISER data set [85]. The equation by Iacumin and Venturelli [66] was used to estimate the drinking water isotope value. The larger group of individuals show calculated δ^18^O_dw_ ranging from -3.7‰ to -6.9‰, consistent with local meteoric water which ranges between -4.1‰ and -6.3‰ as annual average [85]. The number of outliers identified was determined using 1.5xIQR and 2SD statistical methods. The two males and two females whose isotope values fall outside the larger group may come from a warmer, more coastal or possible more arid climate, and from a colder or higher altitude region, respectively. When oxygen isotope data are compared with ^87^Sr/^86^Sr ratios of the same teeth the individuals in the three groups display isotopic values compatible with bio-accessible strontium measured to establish the local signature. Only the female T-24 falls strictly outside the expected strontium range for local origin although she falls into the expected range of the calculated drinking water values for Tauste (Fig 7). Similar values of δ^18^O for Tauste meteoric water showed a broad geographic distribution (Fig 8) overlapping different geological areas. The lack of a strontium isotope composition database in the Iberian Peninsula prevents a determination of the regional provenance of the non-local female T-24.

During Muslim period the Ebro valley was a trade route between Mediterranean coast towards north of the Iberian Peninsula and trans-Pyrenean Europe. Taking on account both oxygen and strontium isotope data the origin areas for these two outliers groups would be confined to the Ebro Valley. Though, during the Early Middle Ages few people regularly travelled because it was simply too difficult and too dangerous. Muslim females were subject to inbreeding marriages so that Tauste outlier females would move by patrilocal marriages. Males would move to get better economic opportunities and possibility of improving status. Tauste non-local males would move from farms to a large village to find new kinds of works. The political instability of the Upper March frontier region also favored displacement of people towards larger and safer urban centers [88].

### Dietary reconstruction

The bone collagen obtained was very well preserved with an average yield of 6.27±4.19%wt (1s.d.). The content of carbon and nitrogen in bone collagen was about 13.1–16.8% and 25.6%–36.2%, respectively, so well-preserved bone collagen should display a carbon/nitrogen molar ratio, based on the content (in %) of these elements in the sample between 2.9 and 3.6 [89,90]. The individual data are given in Table 2.

The δ^13^C ratio for human bone samples ranges between -17.0‰ and -19.9‰ with a mean value of -19.1±0.50‰, and δ^15^N ratios range between 9.6‰ and 17.5‰ with a mean value of 14.9±1.74‰. The rather strong δ^15^N signal of nearly all individuals is noteworthy, with values more than 5‰ over the terrestrial ecosystem baseline (Fig 7), an offset unusually large for a single trophic level effect [91,92,21].

**Fig 9:**
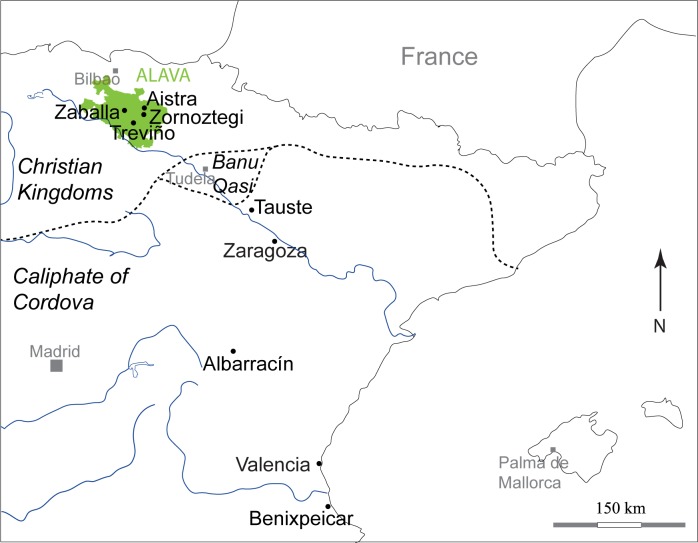
Location of Muslim and Christian archaeological sites and the Upper March or Muslim northern frontier during the 9th century.

**Fig 10:**
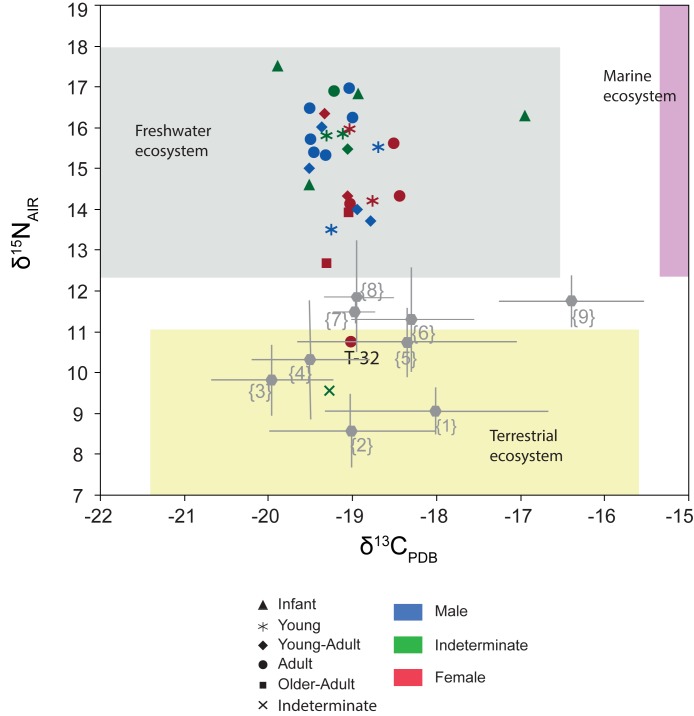
Plot of δ^13^C and δ^15^N values for the individuals at Tauste. Ecosystem boxes are based on faunal data reported by [90] and [92]. Field values are corrected for trophic level effect. 1–9 corresponds to coetaneous Muslim and Christian populations in the Iberian Peninsula: (1) Zornostegui 12th-14th centuries, (2) Aistra 8th-9th centuries, (3) Zaballa 10th-15th centuries, (4) Treviño 12th-14th centuries, (5) Jaca 13th-15th centuries (6) Valencia 14th-15th centuries (7) Albarracín 10th-12th centuries (8) Zaragoza 10th-12th centuries; (9) Benipeixcar 15th-16th centuries (1)-(4) [93], (5)-(8) [94], (9) [95].

Individuals from Tauste were compared with broadly coetaneous Muslim and Christian populations at several locations in the Iberian Peninsula (Fig 9). There are no significant differences in δ^13^C ratios between Tauste (-19.1±0.5‰) and most neighboring Muslim populations (δ^13^C -19.0±0.3‰ in Zaragoza and -19±0.2‰ in Albarracín), or between Tauste and Christian populations (δ^13^C -18.4±1.1‰ in Jaca, -18.4 ±0.6‰ in Valencia, -19.0±1‰ in Aistra, -19.8±0.7‰ in Zaballa, -18.1±1.1‰ in Zornoztegi and -19.6±0.7‰ in Treviño [93–95]) (Table 3, Fig 10). However, the Benipeixcar Muslim population showed enrichment in δ^13^C (-16.36±0.9‰) attributed to marine resource consumption [95]. In contrast, the δ^15^N ratios at Tauste (average 15.0±1.7‰) are unusually high compared with contemporaneous Christian and Muslim populations in the Iberian Middle Ages, whose average values are lower 11‰ (Table 3).

**Table 3:** Mean collagen δ^13^C and δ^15^N of individuals at Tauste and coeval Christian and Islamic sites.

Site	Faith	N	δ^13^C (‰)				δ^15^N (‰)			
			Mean	Std Dev	Max	Min	Mean	Std Dev	Max	Min
**Tauste (8th-10th)**	I	31	-19.1	0.5	-17.0	-19.9	15.0	1.7	9.6	17.5
Male	I	11	-19.2	0.3	-18.7	-19.5	15.5	1	17.0	13.7
Female	I	10	-19.0	0.3	-18.4	-19.3	14.2	1.6	17.5	10.8
Infant	I	4	-18.8	1.3	-17.0	-19.9	16.3	1.2	17.5	14.6
Juvenile	I	6	-19.0	0.3	-18.7	-19.3	15.1	1.0	16.0	13.5
Young adult	I	7	-19.2	0.3	-18.8	-19.5	15.0	1.0	16.3	13.7
Adult	I	11	-19.1	0.4	-18.4	-19.5	15.3	1.8	17.0	10.8
Older adult	I	2	-19.2	0.2	-19.1	-19.3	13.3	0.9	14.0	12.7
Alava (8th-15th)	C	71	-19.1	0.8	-18.1	-19.8	8.7	0.8	9.6	7.9
Zaballa (10th-15th)	C	14	-19.8	0.7	-18.8	-21.3	9.0	0.8	10.4	7.6
Zornoztegi (12th-14th)	C	7	-18.1	1.1	-16.7	-19.9	8.3	0.6	9.2	7.5
Aistra (8-9th)	C	35	-19.0	1.0	16.7	-22.0	7.9	1.0	12.1	6.8
Treviño (12th-14th)	C	15	-19.6	0.7	-18.7	-22	9.6	1.2	12	7.5
Jaca (13th-15th)	C	25	-18.4	1.1	-15.3	-19.6	10.0	0.8	12.2	8.6
Valencia (14th-15th)	C	18	-18.4	0.6	-16.8	-19.3	10.5	1.1	11.7	8.0
Zaragoza (10th-12th)	I	36	-19.0	0.3	-18.2	-19.6	10.9	1.4	14.1	9.0
Albarracín (10th-12th)	I	31	-19.0	0.2	-18.5	-19.4	10.8	0.6	12.1	9.4
Benipeixcar (15th-16th)	I	20	-16.36	0.9	-14.2	-18.0	10.7	0.6	11.9	9.2

C., Christian; I., Islamic. Isotope data of coeval Muslim and Christian sites taken from [93–95].

Faunal samples are required to strengthen conclusions about human diet. Establishing the local isotope composition baseline was problematic since the Islamic burial ritual forbade any objects being buried with the body. Additionally, there is no evidence of Muslim settlement in Tauste to provide coeval fauna. Thus it was not possible to obtain the local baseline with archaeozoological data. Furthermore, present-day local faunal must be discarded because livestock are fed with non-local resources and the isotopic signal will not correspond to local plant resources. Pasture-fed livestock will also exhibit nitrogen isotope depletion due to the widespread use of mineral fertilizers [96,97]. In addition to the fodder and fertilizer effect, livestock trade is another factor affecting isotope composition. All these factors prevented the use of present-day macro-mammals to establish the carbon and nitrogen isotope local baseline.

For these reasons, two local species of small mammals and freshwater fish were analyzed to establish the dietary baseline for Tauste medieval population. Small mammals were selected since present low mobility with restricted home ranges more accurately reflects the local isotopic composition baseline. The analyzed species were wood mice (*Apodemus sylvaticus*), as they are herbivorous (seed eaters), and shrews (*Crocidura russula*), which are one level higher than wood mice in the trophic chain.

**Fig 11:**
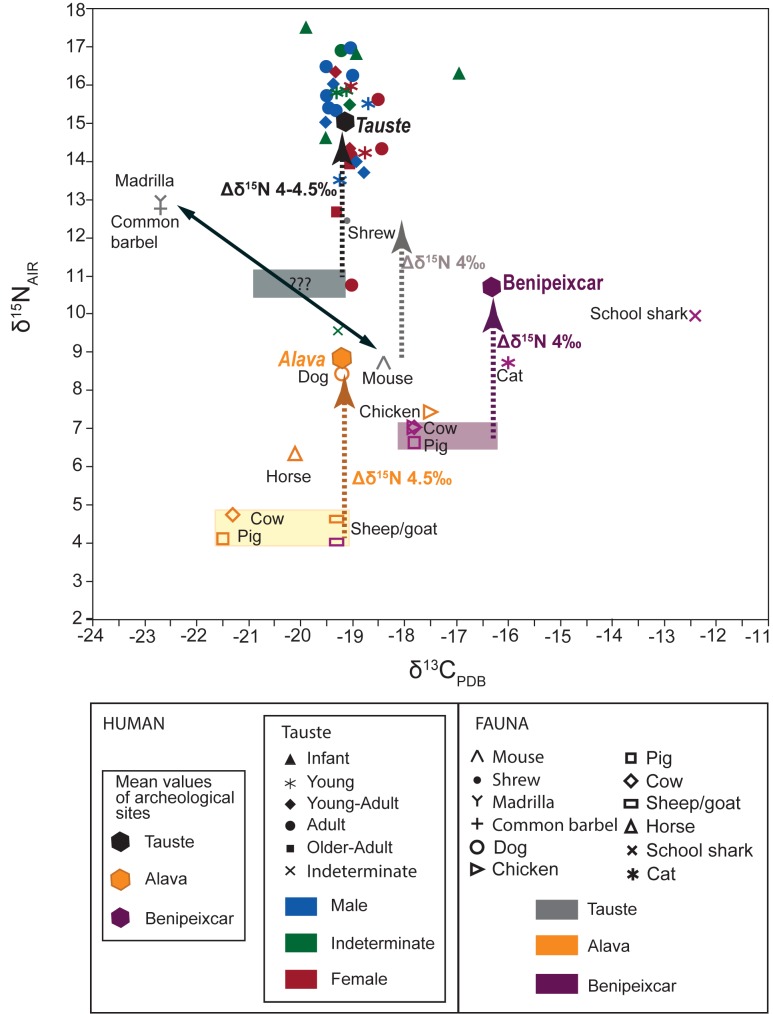
Plot of δ^13^C and δ^15^N values of present-day fauna and medieval human at Tauste and the comparative Alava and Benipeixcar archaeofauna and human dataset [91,93]. Colored rectangles indicate local carbon and nitrogen baseline. Doted arrows show the offset between local baseline and human δ^15^N due to one trophic level enrichment. Grey rectangle indicates estimated local carbon and nitrogen baseline at Tauste.

To address this issue, the isotope enrichment of one trophic level has to be established. For this purpose, both faunal and human values from the nearest coeval Christian and Muslim settlements were compared (Table 4, Fig 11). Livestock from Alava archaeological sites and Benipeixcar Muslim site displayed different average δ^13^C and δ^15^N values. The nitrogen values of plants and fauna are strongly influenced by local climate conditions [98]. Fauna at the Alava sites, located in the northern Iberian Peninsula, displayed lower nitrogen isotope values due to a more humid climate than Benipeixcar, on the warmer and drier Mediterranean coastline.

**Table 4:** Present-day fauna carbon and nitrogen isotope data from Tauste region and archaeofauna data from their coeval sites of Alava and Beneipeixcar.

Site	Specie	N	δ^13^C (‰)				δ^15^N (‰)			
			Mean	Std Dev	Max	Min	Mean	Std Dev	Max	Min
Tauste	Common barbel	2	-22.7	0.3	-22.5	-23	12.7	0.1	12.8	12.7
	Madrilla	1	-22.7	-	-	-	12.9	-	-	-
	Shrew	3	-19.1	2.8	-16	-21.5	12.4	2.2	14.5	10.1
	Wood mouse	3	-18.4	0.9	-18	-19.4	8.7	0.7	9.3	7.9
Alava	Cow	6	-21.3	1.1	-19.8	-22.7	4.7	1.1	6.1	3.5
	Sheep/goat	2	-19.3	0.9	-18.7	-19.9	4.6	2	6.1	3.2
	Pig	3	-21.5	0.6	-20.8	-22.0	4.1	1.9	6.1	2.3
	Horse	1	-20.1	-	-	-	6.3	-	-	-
	Dog	2	-19.2	0.7	-18.6	-19.7	8.4	1.4	9.4	7.4
	Poultry	2	-17.5	2.4	-15.8	-19.1	7.4	0.1	7.5	7.4
Benipeixcar	Cow	5	-17.8	2.8	-14.3	-20.1	7.0	1.2	8.5	5.7
	Sheep/goat	9	-19.3	0.2	-19.1	-19.5	4	0.8	5.6	2.9
	Pig	1	-17.8	-	-	-	6.6	-	-	-
	Poultry	4	-17.8	2.0	-13.3	-17.5	7.0	4.0	9.7	1.0
	Cat	3	-16.0	0.5	-15.5	-16.3	8.7	0.5	9.1	8.1
	School shark	1	-12.4	-	-	-	9.9	-	-	-

Isotope data of coeval Muslim and Christian sites taken from [93,95].

Comparison of human values with fauna revealed the average offset between human and livestock of c. 1.5‰ in δ^13^C and 4–4.5‰ in δ^15^N (Fig 11) typical of one trophic level [99].

In an attempt to establish the trophic level offset in Tauste, small mammal isotope values were considered. The average of wood mice values (-18.4±0.9‰ in δ^13^C and 8.7±0.7‰ in δ^15^N) and shrew values (-19.1±2.8‰ in δ^13^C and 12.4±2.2‰ in δ^15^N) exhibited an offset of c. -1‰ in δ^13^C and 4‰ in δ^15^N. Considering the predictable trophic level offset, the isotope values of the Tauste population should fall within the average of c. -21±1‰ in δ^13^C and 11±0.5‰ in δ^15^N, which is the enrichment typical of one trophic level. Such an enriched δ^15^N baseline can be explained by regional environmental conditions. Regional aridity and the local high relative salinity led to this δ^15^N enrichment [100]. In fact, chemical analysis performed in teeth from Tauste individuals showed the log (Ba/Sr) = -2.35; similar high concentrations of strontium are found in both arid/semiarid region soils and in marine food sources [101,102]. Tauste site is located far from the coastline and the possibility large-scale consumption of marine food can be discarded. Thus Ba/Sr values and the δ^15^N enrichment can be explained by the local bedrock (gypsum and salt) and environmental factors in the Tauste region.

The offset between the isotope ratios of the mice collagen (as equivalent to livestock) and the average of the expected range for human diet suggested a mixture of protein from terrestrial and freshwater sources in their foodstuffs. The relative proportion of terrestrial and freshwater resources in diet can be estimated c. 50–50% using a simple linear mixing model.

However, a particularly high consumption of freshwater fish does not appear justified from either historical or anthropological points of view. Islamic texts about the daily diet in Muslim medieval Spain indicate it was based mainly on cereals: wheat (*Tritium*), barley (*Hordeum vulgare*) and rye (*Secale cereale*), together with such C_4_ grains as millet (*Pennisetum*) and sorghum (*Sorghum*), vegetables and pulses, such as chickpeas (*Cicer arietinum*), lentils (*Lens culinaris*), and peas (*Pisum sativum*), with some regional differences [103–105]. The main sources of proteins were meat and pulses, and the type and quantity of protein consumed varied according to social status and gender. The most highly regarded meats were lamb and poultry. Pork and any animal not slaughtered in a way considered halal were excluded from the diet because Islamic law forbade it. People from lowly backgrounds consumed little meat and often made do with offal because it was cheap. Pulses such as broad beans, chickpeas and lentils constituted another source of protein and were classified as medical food. Although fish was not considered food of great dietetic value, it was part of the diet of the people from more humble backgrounds, particularly in river or coastal areas [106].

Another possible interpretation for the high δ^15^N enrichment in the Tauste population is to consider the manuring effect on plants. Since the use of manure was an advanced agricultural method introduced by Muslims in the Iberian Peninsula [107] all plants from manured soils showed a δ^15^N enrichment that can be about 5‰ in cereal δ^15^N [30]. Consequently, domestic animals foddered with manured chaff and grains will present higher δ^15^N values. Assuming the consumption of plants enriched in δ^15^N by the manure effect, the contribution of freshwater resources may decrease and become less than 10% of dietary protein intake.

Another interesting aspect of the reconstruction of the Tauste inhabitants’ palaeodiet is the variation in isotope composition by sex and age. For the comparison, female T-32 was excluded because her strontium isotope composition indicates a return to Tauste in the last years of her life preventing δ^15^N and δ^13^C remodeling to the local isotope signature. The mean values of the male samples are 15.5±1.00‰ for δ^15^N and -19.2±0.31‰ for δ^13^C. In female samples, the mean values are 14.6±1.15‰ for δ^15^N and -19.0±0.32‰ for δ^13^C. Comparison of the results with the Student's t-test for males and females confirms the significant differences in δ^15^N (t_18_, p = 0.026). These differences suggest that females and males had different access to animal protein, probably due to the sexual division of labor [108]. In fact, written evidence suggests the diet of Muslim females and males differed. Medical treatises make recommendations about diet, and special recommendations were made for females during pregnancy or lactation [109]. Two meals a day were recommended but in practice a smaller meal as breakfast was taken by working males. Lower dietetic needs were expected of females since female labor was restricted to domestic tasks and other activities in the household [110]. However, the differences in nitrogen composition related to sex are determined by the middle-aged adult male (ages 35–50 years) composition. In fact, excluding middle-aged adult males differences between sexes are not significant. Middle-aged adult males show higher δ^15^N values than young males and females of any age. Variations between adult males and the other age segments of population indicate a different dietary intake which may be related, for instance, to a greater meat protein consumption.

Finally, the highest δ^15^N values were found in children younger than 3 years, whereas children older than 4 years old have similar δ^15^N values to those of adults. In younger infants, higher δ^15^N values are due to a “nursing effect” indicating a diet based mainly on maternal milk.

However considering the significantly wide chronological span and the small number of radiocarbon dates, it is not possible to rule out temporal dietary differences, which is to say that there may have been variations in the diet in the course of time.

## Conclusions

Isotope signatures in archaeological human remains have been used to investigate palaeomobility and the palaeodiet of the medieval Muslim population at Tauste, on the changing frontier between Muslim and Christian kingdoms in the 8th to 10th centuries.

The combination of strontium and oxygen isotope analyses was able to discriminate non-local and local individuals. Although Tauste was located on the northern al-Andalus frontier most individuals were of local origin and only three females and two males were non-locals. Establishing the provenance of incoming individuals is difficult as strontium isotope ratios indicate a similar geological region. According to the oxygen isotope composition, two males would come from a warmer region while two females would come from a more mountainous geographical area. Also T-24 was of non-local origin since the different strontium values indicate a different geological provenance. Within the local population, the female T-32 stand out because she was born in Tauste, some years later she moved and lived in another geological region and then returned to Tauste a few years before she died.

As regards the medieval Muslim diet, the δ^13^C and δ^15^N results illustrate not only differences in diet according to sex and age but also the environmental conditions. The extremely high δ^15^N values in Tauste population (δ^15^N = 15.0‰ on average) indicate an anomalously high δ^15^N baseline that can be explained by the concurrence of (1) geological and environmental conditions, (2) the manuring effect on vegetables, cereals and livestock and (3) the consumption of freshwater fish. The amount of fish in the diet varies from 50% to <20% as the manuring effect increases. Significant differences were observed in δ^13^C and δ^15^N by sex, indicating different diets that may be related to the sexual division of labor since Muslim female work was restricted to the household. The main dietary differences between males and females were amongst adult individuals, suggesting adult males were differentially valued in medieval Muslim society and consumed more animal protein than females and young males. The lower δ^15^N values of the elder females indicated lower protein consumption due to lesser dietary needs. In contrast, the significant higher δ^15^N values in the children younger than 4 years could be related to the “nursing effect”.

## Supporting information

S1 Table Radiocarbon data with the 1 sigma and 2 sigma calibrations. Dating of human bone samples from Tauste Muslim necropolis was carried out in Roca Solano Laboratory (CSIC Madrid).
(XLSX)

S2 Table Strontium, oxygen, carbon and nitrogen isotopes analyses on human samples. (XLSX)

S3 Table Strontium isotope analyses on water and soils samples used as comparative data for the local range at Tauste. (XLSX)

S4 Table Results of the carbon and nitrogen isotope analysis of the present-days faunal remains from Tauste used as comparative data for the local isotope baseline. (XLSX)
